# Short-beaked echidna (*Tachyglossus aculeatus*) home range at Fowlers Gap Arid Zone Research Station, NSW

**DOI:** 10.1371/journal.pone.0242298

**Published:** 2021-04-16

**Authors:** Georgia J. Badgery, Jasmin C. Lawes, Keith E. A. Leggett

**Affiliations:** 1 School of Biological, Earth and Environmental Sciences, UNSW Sydney, Sydney, New South Wales, Australia; 2 Fowlers Gap Arid Zone Research Station, NSW School of Biological, Earth and Environmental Sciences, UNSW, Fowlers Gap, New South Wales, Australia; 3 Centre for Ecosystem Science, School of Biological, Earth and Environmental Sciences, UNSW Sydney, Sydney, New South Wales, Australia; 4 Surf Life Saving Australia, Sydney, New South Wales, Australia; Sichuan University, CHINA

## Abstract

Echidnas *(Tachyglossus aculeatus)* are found Australia-wide and appear to be remarkably well-adapted to the arid zone, yet nearly all echidna research has been conducted in temperate, tropical and alpine zones. This study investigated the home range and movement of echidnas in western New South Wales. Radio telemetry tracking was used to locate the echidnas daily during the study period (March-May 2018, November 2018, March-May 2019 and August 2019); the observed home range was 1.47± 1.21km^2^. This is over twice the reported home range of temperate environments (<0.65km^2^), suggesting that echidnas exhibit larger home ranges in arid zones. The home range of individual echidnas ranged from 0.02km^2^ to 3.56km^2^. Echidnas exhibited a small degree of overlap (6.6%± 19.8%) but this varied considerably between individuals (between 0 to 84.2% overlap.) Four out of the thirteen echidnas died during this study, likely due to the severe drought that occurred during the study. This study provides insight into the movement and home range of echidnas in arid zones, revealing that desert echidnas have large home ranges, probably dependent on the availability of resources.

## Introduction

Over the past 200 years, Australian terrestrial mammals have suffered a population decline over 90% [1], additionally, over 30% have become extinct or face possible extinction, primarily due to land clearing and the introduction of exotic species [1, 2]. Medium-sized mammals (34-4200g) have been the worst affected, with a 25% loss [2, 3]. One exception is the short-beaked echidna (*Tachyglossus aculeatus*), which has shown little to no range or population declines despite habitat loss and fragmentation [2]. Echidnas are not restricted by habitat, and although they are found to readily utilise fallen logs and leaf litter for shelter when available, vegetation is not necessary, allowing them to survive outside of nature reserves [2]. Echidnas are specialised to eat ants and termites which are widespread across all climate and habitat types, inferring that echidnas are unlikely to be restricted by resource availability [2]. Echidnas are also able to tolerate extreme Australian environmental conditions including drought and fire due to their low metabolic rate [2]. While foraging, echidnas act as environmental engineers, excavating large amounts of soil and leaf litter [4]. This process turns and aerates the soil, increasing its fertility [5]. Foraging pits are essential resource-sinks in arid zones, so understanding their foraging habits in these zones is essential to understanding the role of echidnas, especially in arid areas where resources are scarce [5]. Echidnas are likely to be more ecologically important in arid zones than ecological zones that receive higher rainfall as they contribute to a larger percentage of the biota [6].

Animals move and migrate for many reasons; to find food, shelter or mates or to avoid predation and competition. However, their movements are not random, rather, they are restricted to defined areas, or home ranges [7]. This paper defines home range as the area used predictably and regularly for daily metabolic needs [8, 9]. Home range research has greatly extended our understanding of the ecology and behaviour of animals [7], and generally involves tracking individuals using a locational device (i.e. GPS or radio-telemetry). Although radio-telemetry involves the observer following and locating the tagged animal, it is a reliable method of tracking animals that do not have large ranges, and it is also significantly cheaper than remote-tracking using GPS. The versatility of radio-telemetry tracking means it has become central in studying the ecology of wildlife [10, 11]. This tracking technique has been used to investigate the home range and daily movements of animals globally, including African elephants (*Loxodonta africana*) [11], vultures, condors [10] and invertebrates such as the tiger spiketail dragonfly (*Cordulegaster erronea*) [12].

Both minimum convex polygon and kernel analysis have been widely used for home range analysis [8, 9] despite their marked difference in calculation techniques. The minimum convex polygon technique draws the smallest possibly polygon around all the points in the home range, ensuring that the interior angles do not exceed 180 degrees. Minimum convex polygons are easy to conceptualise and do not rely on a specific statistical distribution, however, it often encompasses areas that are never or rarely used by the animal [13]. Kernel analysis uses the density of points to create a statistically weighted home range [13]. Kernels can quantify the intensity of space use, estimating the likelihood of finding an animal at particular locations. However, the home range size is heavily affected by the smoothing factor which may cause inconsistencies. Both methods have their strengths and weaknesses, so this study uses both to try to accurately estimate the home range of echidnas in an arid zone [7, 11].

Echidnas are reported to remain within a defined home range if the habitat is suitable [14]. Radio-telemetry tracking was used early in echidna research to calculate the home range of echidnas on Kangaroo Island [14]. This study used the circle method of home range calculation, where the furthest distance between two sightings of the same echidna is used as the diameter of a circular home range [14]. This study was conducted on Kangaroo Island and reported an average home range of 800m in diameter (approx. 0.5km^2^). However, this method of calculation was found to give gross overestimations of echidna home range [15]. The home range and movement ecology of echidnas has since been studied in semi-arid Western Australia [16], temperate Tasmania [17], sub-tropical Queensland [8] and the sub-alpine Snowy Mountains [15]. The reported home range of these echidnas were 0.65±17km^2^, 0.61±20.5km^2^, 0.50±0.25km^2^ and 0.42±0.20km^2^ respectively [8, 15–17]. These studies used direct calculation methods and minimum convex polygons which are more sophisticated methods of home range calculation than the circle method described before. These studies also demonstrated a large overlap of conspecific home ranges and strengthened the argument that adult echidnas remain in their home range their entire life.

There is, however, limited information on echidna movement in arid zones despite these areas making up 70% of Australia’s land area [18]. These ecosystems generally have lower biodiversity and scarcer, patchier resources than higher rainfall areas, hence animals often have to travel further in order to meet their energy requirements [6]. In arid environments, productivity is greatest in the riparian zones; where aquatic ecosystems transition to terrestrial ones [19]. In arid zones, the riparian ecosystems are often comprised of ephemeral flood out zones, where the surface water is low, unpredictable and irregular [19]. Accordingly, fertile areas are concentrated in patches amongst areas of extreme infertility [20].

This study will investigate the home range and daily movement of echidnas in an arid zone and compare this to previous studies of echidna home range in more temperate regions. This will provide an insight into the trends of home range in different climatic zones. Since resources are scarcer and patchier, we hypothesise that home ranges will be larger in arid environments (where resources are scarce) compared to higher rainfall areas (where resources are relatively abundant).

## Materials and methods

### Study site

This study was conducted at Fowlers Gap Arid Zone Research Station (31°05’S, 141°43’E), 110km north of Broken Hill, NSW between March 2018 and September 2019. The station was established in 1966 by the University of New South Wales as both a research and working sheep station [21]. The climate at Fowlers Gap is arid, with a 50-year mean annual rainfall of 230.7mm, although this is highly variable [21, 22]. In summer, daily temperatures exceed 30°C, while the temperatures in winter are mild, rarely falling below 0°C [21].

During and for 2 years prior to this study, Fowlers Gap had experienced a drought with annual rainfalls of 84.4mm in 2017, 48.2mm in 2018 and 42.0mm in 2019 [22]. Arid zones are particularly susceptible to climatic changes, and this prolonged drought is likely to have altered the landscape and biodiversity of the region [6].

The study area covered 15km2 around the main homestead, encompassing Fowlers Creek, Homestead Creek and Gum Creek and an earthen dam known as “Lake”. The area is characterised by arid shrubland, predominately saltbush (*Atriplex vesicaria*) [23, 24], while perennial vegetation is mostly absent. River red gums (*Eucalyptus camaldulensis*) and prickly wattle (*Acacia victoriae*) dominate the riverine woodlands, resulting in a large build-up of leaf litter that can be used by echidnas for shelter as well as food for their prey [23, 24]. The ephemeral creek habitats are characterised by steep, well defined banks surrounding narrow flow beds as well as and sparse trees both in the flow bed and along the bank shelves. The earthen tank habitat consists of a semi-permanent water hole surrounded by indistinct banks, ephemeral flow bed and flood out. The bank and ephemeral flood out area is covered with river red gum and a dense layer of leaf litter which creates a cool, moist environment [19]. The only known successful predator of adult echidnas, the dingo (*Canis lupus dingo*) is absent from this study site [2].

### Home range analyses

Tx-VHF Transmitters (Model F1840) were fitted to ten echidnas in 2018 and three more echidnas were tagged during the study, in April 2019. These transmitters were manufactured by Advanced Telemetry Systems. They have a battery life of 787 days and send 40 pulses (between 150-152MHz) per minute (ppm).

Echidnas were found by searching throughout the study area (around the Folwers Gap Homestead) while the animals were foraging or moving. Search effort was evenly distributed across the entire study site. Once echidnas were located, spines on the acnestis were clipped to ~5 mm long, and a 20 g transmitter was attached using a fast-setting non-toxic epoxy resin (Gorilla Glue Inc. USA). The transmitters did not exceed 5% of the smallest echidna’s body mass, which is the recommended limit [25]. Each echidna was weighed upon capture and categorised as either juvenile (<2 kg) or adult (>2 kg) [26]. Echidnas were identified as females if there was an absence of hind-leg spurs [17] as other sex-specific characteristics such as pouch development in females, or penis protrusion in males were not reliably identified [17, 27].

Over a 2-year period, thirteen adult and sub-adult echidnas were tracked to calculate home range of echidnas at Fowlers Gap. The echidnas were numbered: E1-13, with number 1 being the earliest tagged, on 16 March 2018 and number 13 being tagged last, on 27 April 2019. Of these, E1, E2, E4, E5, E6 and E13 were reliably identified as females, based on the absence of hind leg spurs [17]. Weights of echidnas, at the time of capture, ranged from 1.1–4.8kg. E1 and E4 were found dead on 29/11/18, E7 and E8 were found dead on 22/2/19. Although it was not possible to determine exactly when the animals died, the amount of decay suggests none had been dead for longer than a few weeks. No signal could be detected from E2 or E5 during the 2019 but no carcasses were recorded so it is assumed that their tags failed early. No signal was located for E9 and E12 during September 2019 (assumed to be tag failure). E13 lost her radio tracker sometime between May and August 2019.

Echidnas 1–10 were located regularly during March, April, May and November 2018 part of a prior study. E3, E6, E0, E10, E11, E12 and E13 were located regularly during March, April and August 2019. Echidnas were located daily using a VHR receiver (ATS R2000) and a handheld 3 Element Folding Yagi Antenna (ATS). Searching commenced on hills or creek banks near the last recorded location of the animal and continued until reliably located the echidna by either: physically observing the echidna or finding a pin-pointed signal above a burrow. Once located, the foraging or resting site was marked with yellow flagging tape and the geographical coordinates were obtained using a Garmin handheld GPS with at least 4 m accuracy. If a signal could not be located after checking from all peaks in and around the study area, it was assumed that the echidna was buried too deep or was hiding in a gully or rock cave where the signal was blocked.

As with previous echidna home range studies, [8], independent locations were determined by:

1. All locations of echidnas not in shelters, or
2. All locations of echidnas in shelters, unless they have previously occupied that shelter with no evidence that they have left the shelter and then returned.

Previous research has suggested when using statistical techniques to predict home range, at least 15 independent data points were required to best reflect the true home range of the animal [15]. Hence, animals with less than 15 independent data points (E7, E8, E12 and E13) were not used for home range analysis.

This study was undertaken on research permit (Approval number: 18/3B) issued by the UNSW Animal Ethics Committee, and NSW National Parks and Wildlife Service scientific license number: SL102050.

### Analyses

Home range of each echidna was mapped using R and ArcMap 10.8. The base map was provided by © State of New South Wales (Spatial Services, a business unit of the Department of Customer Service NSW) under the CC-BY 4.0 licence (spatial.nsw.gov.au). Home range of the echidnas was estimated by minimum convex polygon (MCP) analysis (95%) using the *Minimum Bounding Geometry* [8, 9]. The kernel function in R was used to further analyse home range. The fixed kernels used during this analysis were 50% and 95% of loci, which correspond to ‘core area’ and ‘peripheral area.’ The smoothing factor of the kernel was adjusted so that the area of the 95% kernel was approximately equal to the area of the MCP, although this was not possible when echidnas appeared to leave their home range [13].

## Results

Table 1 summarises the data collected from the nine echidnas that were used for home range analysis including capture weight and sex. The home range of echidnas at Fowlers Gap (95% MCP) ranged between 0.02km^2^ to 3.65km^2^. The peripheral areas (95% kernel) ranged between 0.31km^2^ to 1.87km^2^ while the core areas (50% kernel) were between 0.04km^2^ to 0.38km^2^ (Table 1). The 95% kernel was much smaller than the 95% MCP for echidnas E4 and E6. However, the 95% kernel was much larger than the 95% MCP for E5. Seven out of nine of the echidnas shared a home range with at least 2 other echidnas.

**Table 1:** Home ranges of echidnas determined by three different calculation methods: 95% kernels (peripheral area), 50% kernels (core area) and 95% minimum convex polygons (MCP).

Echidna ID	Weight on capture (kg)	Number of independent locations	Kernel (km^2^)		MCP (km^2^)	Number of conspecifics within the home range
			50%	95%	95%	
E1	1.2 F*	19	0.16	0.79	0.68	0
E2	2.9 F	41	0.12	0.54	0.44	2
E3	4.02	73	0.23	1.42	1.20	5
E4	2.9 F*	16	0.08	0.50	3.56	0
E5	1.1 F	32	0.04	0.31	0.02	2
E6	2.1 F	90	0.38	1.87	3.38	6
E7	1.1 F*	4	-	-	-	-
E8	4.8*	11	-	-	-	-
E9	2.8	59	0.17	0.93	0.79	6
E10	2.3	59	0.13	0.61	0.36	2
E11	3.5	21	0.24	1.05	1.01	6
E12	3.3	4	-	-	-	-
E13	3.1 F	6	-	-	-	-
Mean			0.17	0.89	1.27	
SD			0.10	0.49	1.29	

“F” indicates that the echidna was positively identified as female.

‘*’ indicates that the animal was confirmed dead during the field study. SD: Standard deviation. Home range was not calculated for animals with less than 15 independent locations.

**Fig 1:**
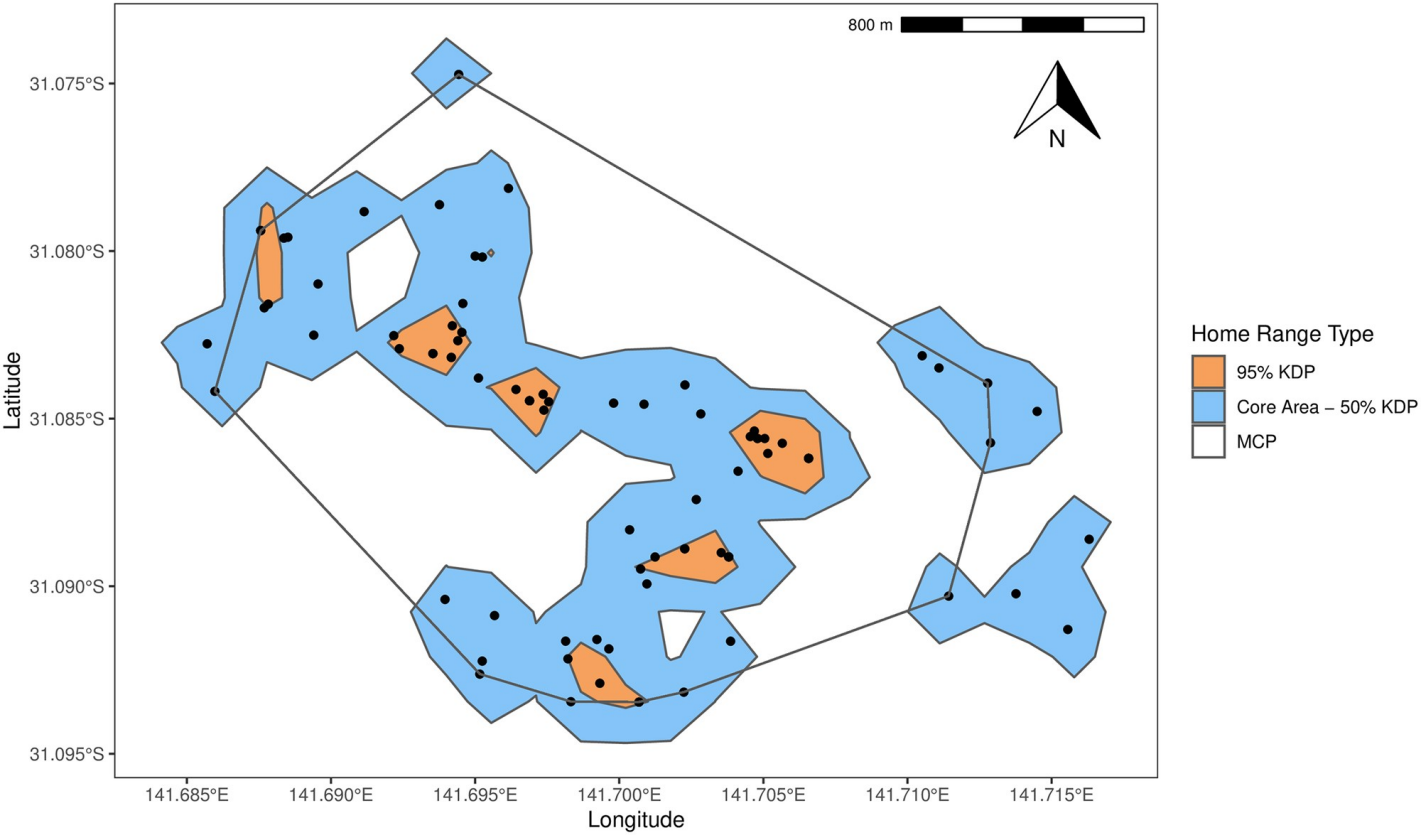
Minimum convex polygon, 95% kernel and core area of E6 including individual data points.

Fig 1 shows the difference between MCP and kernel analyses using the E6 as an example. The 95% kernel is fragmented, displaying three distinct clusters that extend beyond the individual data points which reflects where an animal spends 95% of its time. The MCP creates one distinct shape that encompasses all the individual data points.

**Fig 2:**
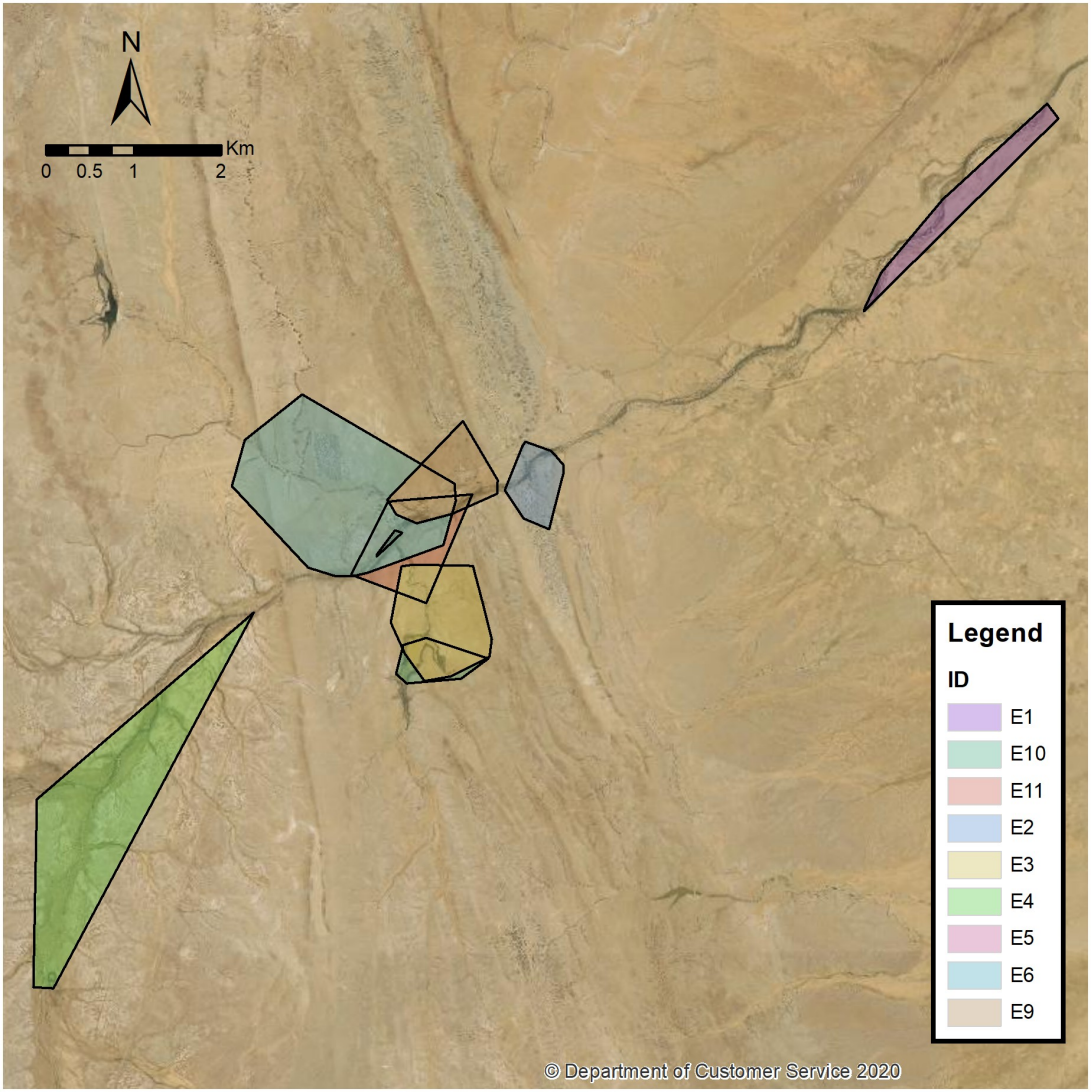
Home range of echidnas E1, E2, E3, E4, E6, E9, E10 and E11 using MCP. The base map was reprinted from the © State of New South Wales (Spatial Services, a business unit of the Department of Customer Service NSW). For current information go to spatial.nsw.gov.au.

Fig 2 maps the MCP of eight echidnas (E1, E2, E3, E4, E5, E6, E9, E10 and E11). E2, E3, E5, E6, E9 E10 and E11 are clustered around the Fowlers Gap Homestead while E1 and E4 spread out to the north and south (respectively) of the homestead.

Table 2 shows the percent overlap between the home ranges of two echidnas. The percentage overlap between home ranges was found to vary between zero and 84.2% (mean = 6.61%, SD = 19.8%). E5 has a small home range which is 100% included in the home ranges of E6 and E11, while E5 only makes up 2 and 10 percent of E6 and E11’s home range, respectively. Table 3 compares the home ranges of echidnas at Fowlers Gap to that of other areas.

**Table 2:** Percentage overlap of echidna home ranges.

	Echidna ID						
		**E3**	**E5**	**E6**	**E9**	**E10**	**E11**
Echidna ID	**E3**	-	0	0	0	**64**	0
	**E5**	0	-	**2**	**3**	0	**10**
	**E6**	0	**100**	-	**39**	0	**43**
	**E9**	0	**50**	**9**	-	0	**12**
	**E10**	**16**	0	0	0	-	0
	**E11**	0	**100**	**13**	**15**	0	-

**Table 3:** Comparison of the home range size of echidnas within different climate zones.

Location	Climate Zone (30 year annual rainfall mean)	Home Range (mean ± SDkm^2^)	Reference
**Fowlers Gap, New South Wales**	Arid (227mm)	1.27±1.29	This study
**Western Australian Wheat Belt**	Semi-Arid (340mm)	0.65 ± 0.17	Abensperg-Traun (1991)
**Flinders Chase National Park, Kangaroo Island, South Australia**	Sub-Tropical (534mm)	0.50±0.01	Augee et al. (1975)
**Southern Midlands of Tasmania**	Temperate (475mm)	0.61 ± 0.21	Nicol et al. (2011),
**South-Eastern Highlands of Queensland**	Sub-Tropical (767mm)	0.50 ± 0.25	Wilkinson et al. (1998)
**Snowy Mountains, New South Wales**	Sub-Alpine (1275mm)	0.42 ± 0.20	Augee et al. (1992) Griffiths (1978)

## Discussion

The home range of echidnas at Fowlers Gap is comparatively very large, being on average 1.27 km^2^, twice as large as home ranges that reported in semi-arid Western Australia (0.65 km^2^) [16]. The larger home range is likely due to the sparser resources in the arid zone compared to areas of higher rainfall, forcing echidnas to travel further to obtain sufficient resources [6]. Home range of the majority of the tagged echidnas at Fowlers Gap was concentrated around riparian zones, likely due to the larger amounts of vegetation, and hence, greater shelter and prey availability [26]. Since the relationship between energy needs and food availability are known to contribute to home range size, it is likely that at Fowlers Gap echidnas needed to utilise larger home ranges to meet their energy requirements [28, 29].

Echidnas are thought to have a stable range that they occupy throughout their lives [14] however, during this study, three adult echidnas could not be found after being tagged in the study area. It is assumed that these echidnas dispersed from the study area, as no signal or carcasses were found after extensive searching. Dispersals are most commonly seen in young males [30], but since all the juveniles from this study were female, it is believed that drought conditions during this study were a greater dispersal factor than age or sex [2]. Drought conditions may have caused previous home ranges to become too resource-poor, forcing animals to seek out new areas with more available resources. Hence, it appears that the resource availability could be a factor in determining echidna home range size and movements. E5 appeared to leave its home range and was not able to be located after May 2018, this could account for the disparity between the home ranges recorded by the 95% MCP (0.02km^2^) and the 95% kernel (0.31km^2^). It is possible that E5’s dispersal was due to resource availability as its movement range overlapped 100% with 2 echidnas and 50% with another. E5 was a juvenile (1.1kg) that had likely recently been weaned and needed to establish its own home range elsewhere.

The home ranges of echidnas at Fowlers Gap varied between individuals. E1, E2 and E10 that displayed a home range similar in size to that of sub-tropical and sub-alpine zones where resources are abundant [6, 8, 15] (Table 3). The home range of E10 was concentrated around an earthen tank suggesting that this area may be more resource-rich compared to the creeks and non-riparian zones. Hence, the lake habitat appears to provide a superior habitat to that of the creek beds and non-riparian zones. Non-riparian zones are reported to be more resource-poor than riparian zones [20], this could explain why E10 had a smaller home range than echidnas that were based around non-riparian zones. However, it is not clear why the home range around creeks is larger than around the earthen tank. It is possible that the earthen tank and watering points may support a higher carbon concentration (more trees, leaf litter and detritus), and hence be more resource-rich than the natural ephemeral creeks [20].

E4 and E6 exhibited relatively large home ranges (Table 1). E4 appeared to disperse and leave its home range before being found dead in November 2018, this likely accounts for the large MCP home range calculation, potentially the 95% kernel analysis provides a more accurate account of E4’s home range. This dispersal of a female, adult echidna was possibly due to a lack of resources forcing it to seek a new home range. However, E6, an adult female, consistently utilised its home range for the entire duration of this study. This echidna was mostly found along two creek lines and associated banks and flood out areas. 43% of E11’s home range overlapped with that of E6, sharing a resource rich area around the homestead and along a creek. However, E11 demonstrated a significantly smaller home range than E6. It is unclear as to why E6 used such a large home range when it shared a resource rich area with E11. Previous research has suggested that, like most solitary mammals, male echidnas have larger home ranges than female echidnas [26, 31]. However, since E6 is a female, sex is unlikely a contributing factor to the smaller home range of E11. The two largest home ranges were that of female echidnas. There has not been a consensus about a relationship between home range and body mass, with only one previous study noting a significant positive relationship [8]. On capture E6 was 2.1, kg while E11 was 3.1, so if there was a positive relationship between body mass and home range size, it would be assumed that E11 would have the larger home range. Hence, the drought and reduced resource availability is likely a driving factor for echidna home range, with E6 needing to travel further in order to have enough food available, especially when sharing foraging areas with conspecifics. Further research into these shared home ranges and the interaction between conspecifics would be valuable to the understanding of echidna movement and ecology.

This study showed that echidnas had a smaller degree of overlap of home ranges than in previous studies, an average overlap of 6.61% compared to 24% in south-eastern Queensland and extensive overlap in the Snowy Mountains [8, 15]. The more sparsely distributed resources in arid zones, suggests that there may not be enough food for echidnas to share home ranges. Since echidnas do not use consistent shelters or tracks and they are not attracted to baits or recordings [30], it is also possible that this lack of overlapping home range is because there are more echidnas in the area that have not yet been tagged. Despite extensive searching, it is impossible to know how many echidnas are in an area, and given that new echidnas continued to be found in 2019 suggests that there may be a larger population than initially believed.

At least 30% of the tagged echidnas (four animals) died during this investigation, and another 30% could not be located continuously during study. The largest (4.8kg) and one of the smallest (1.1kg) echidnas, were among those confirmed dead, with the other young echidna (1.1kg) appearing to disperse. There was no clear cause of death for any of the four echidnas found dead, and there was no dispersal of carcass parts, indicating that they were not predated. All of the echidnas found dead had been tagged at least six months prior to their death, this, as well as no adverse effects reported in previous research suggests that the tags/tagging process are highly unlikely to have caused their deaths. None of the deceased animals presented with obvious low body condition prior to being found dead, although there was a period of a couple of months between when they were last sighted alive, excluding E8 who had been located a week before. This suggests that the health of these animals may have declined very quickly, coinciding with the onset of a severe drought which probably impacted the availability of food. E1 and E7 were likely juveniles, and they may not have been able to establish a suitable home range resulting in them being less familiar with the location and quality of feeding and shelter sites.

Echidnas do not have sweat glands, nor do they pant, meaning that they rely on behavioural thermoregulation in order to keep their body temperature under the lethal limit of >38°C [14]. So, while echidnas do not normally rely on shelter, in the extreme heat of the 2018/19 drought, knowing when, where and what kind of shelter to retreat to could be vital to their survival. Studies have shown that juveniles do not show a preference for shelter, suggesting they may not have the knowledge to survive extreme heat, for example, logs have been found to be 1–2°C cooler than ambient temperature [8]. E8 however, was the largest and potentially the oldest echidna, so it is unclear as to why it may have died. The drought overall would have had adverse effects to all animals with reduced food availability, shelter quality and increased heat.

This study has provided data that suggests that echidna home range is much larger in arid zones compared to semi-arid, temperate, subtropical and sub-alpine zones. The death of 30% of the tagged echidnas and the dispersal of adults suggests that these echidnas were adversely affected by the drought, struggling to find recourses and shelter. While other studies found a large overlap of conspecific home range, this study found reduced overlap of home range, likely due to the reduced resource availability in an arid zone.
